# Supplementary material for: Ni-Catalyzed Enantioselective Intramolecular Mizoroki–Heck Reaction for the Synthesis of Phenanthridinone Derivatives
Source: J Org Chem. 2023 Jun 15;88(13):8203–26. doi: 10.1021/acs.joc.3c00202 (PMC10337041; doi:10.1021/acs.joc.3c00202)
Supplement: Supplementary file 2 — jo3c00202_si_002.zip [file jo3c00202_si_002.zip › Chiral ligands/4a 6F-iQ-NO/19F 6F iQ N-O 4a/3/pdata/1/email_DR-117-078 19F_3_1.pdf]

DR-117-078 19F
F19 CDCl3 {D:\nmrusers\malachowski} BM 18

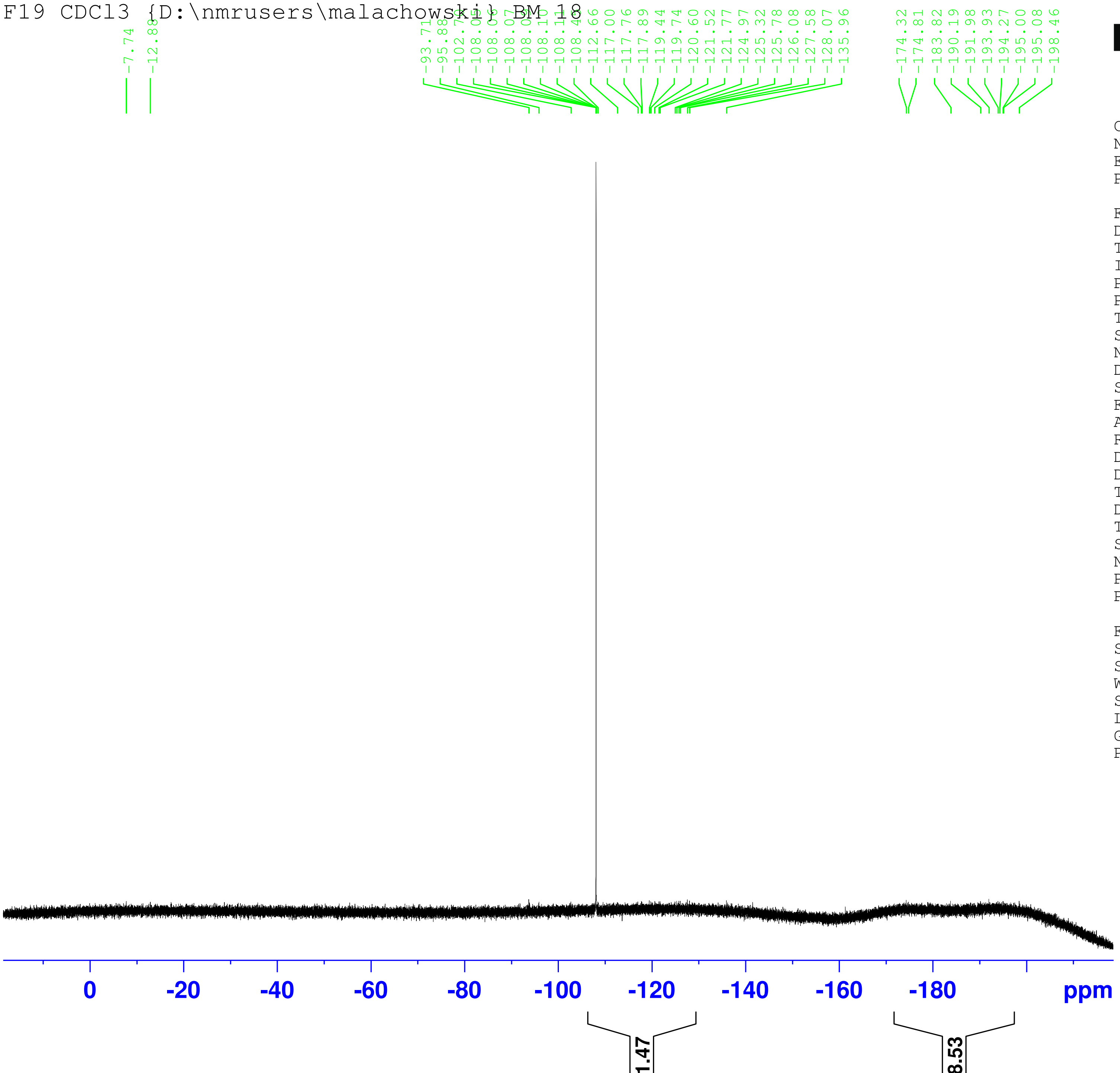

Current Data Parameters
NAME DR-117-078 19F
EXPNO 3
PROCNO 1

F2 - Acquisition Parameters

Date_	20220815	
Time	10.27	h
INSTRUM	spect	
PROBHD	Z104450_0352 (	
PULPROG	zgflqn	
TD	131072	
SOLVENT	CDCl3	
NS	16	
DS	4	
SWH	89285.711	Hz
FIDRES	1.362392	Hz
AQ	0.7340032	sec
RG	203	
DW	5.600	usec
DE	6.50	usec
TE	298.0	K
D1	1.00000000	sec
TD0	1	
SFO1	376.4795333	MHz
NUC1	19F	
P1	12.30	usec
PLW1	19.19499969	W

F2 - Processing parameters

SI	65536	
SF	376.5171850	MHz
WDW	EM	
SSB	0	
LB	0.30	Hz
GB	0	
PC	1.00	
